# Two Cases of Death after Extraction of Teeth

**Published:** 1884-06

**Authors:** 


### Two Cases of Death after Extraction of Teeth.

In the *Vratch*, No. 34, p. 523, Dr. V. M. Zakharevitch details the cases of two strong and generally healthy medical men, aged 26 and 24, who died, one on the sixth and the other on the tenth day after extraction of teeth. In each of the patients the left inferior second molar tooth was removed. In one of the cases, osteomyelitis, and in the other, osteitis and periostitis of the lower jaw developed, all the symtoms being unusually severe. The author believes that the inflammation from its onset was of a septic character, the wound being septically poisoned by the dentist having employed unclean instruments. In view of these two exceedingly sad cases, Dr. Zakharevitch strongly insists on the necessity of strict antiseptic precautions in tooth-extraction. He makes his patients, before the operation, carefully cleanse their teeth by means of a brush with plenty of soap, and wash out the mouth with one or two per cent. carbolic solution. The carbolic washing is repeated after extraction. When the bleeding is arrested, he powders the wounded alveolus with iodoform, puts into its cavity a small plug of wadding, sprinkled also with iodoform and seals the socket with collodion. All the instruments employed are disinfected both before and after the operation.—*London Medical Record.*

